# Toxidermie médicamenteuse sous hydroxychloroquine pour traitement systémique d’un syndrome de Gougerot-Sjögren primaire: à propos d’un cas

**DOI:** 10.11604/pamj.2021.38.283.28478

**Published:** 2021-03-18

**Authors:** Ansumana Mohammed Keita, Mouna Zahlane, Laila Benjilali, Lamiaa Essaadouni

**Affiliations:** 1 Service de Médecine Interne, Hôpital Arrazi, Centre Hospitalier Universitaire Mohammed VI, Marrakech, Maroc

**Keywords:** Exanthèmes maculo-papuleux, hydroxychloroquine, syndrome de Gougerot-Sjögren primaire, à propos d’un cas, Maculopapular rashes, hydroxychloroquine, primary Gougerot-Sjögren syndrome, case report

## Abstract

Nous rapportons un cas d´exanthème maculo-papuleux apparu après une semaine de prise de l´hydroxychloroquine 400mg prescrite pour une atteinte articulaire d´un syndrome de Gougerot-Sjögren primaire chez une patiente de 41 ans. Cette dernière était suivie pour une lésion glomérulaire minime idiopathique depuis plus d´un an, traitée efficacement avec la corticothérapie. Les exanthèmes maculo-papuleux ont régressé après avoir reçu l´hydrocortisone, la desloratadine et l´arrêt immédiat de l´hydroxychloroquine. Notre cas reflète l´importance d´insister sur la prescription à faible dose de l´hydroxychloroquine chez les sujets avec des pathologies préexistantes rénales et aussi l´intérêt de sensibiliser et d´éduquer les patients des effets secondaires de l´hydroxychloroquine.

## Abstract (en)

We here report the case of a 41-year-old female patient with maculopapular rash occurring a week after receiving hydroxychloroquine 400 mg for primary Gougerot-Sjögren syndrome with articular involvement. The patient had more than 1-year history of idiopathic minimal glomerular lesion, effectively treated with corticosteroid therapy. Maculopapular rashes resolved after hydroxychloroquine treatment was stopped and the patient was given hydrocortisone and desloratadine. Our case highlights the importance of prescribing low dose hydroxychloroquine in subjects with a history of kidney disease as well as of raising awareness and educating patients about side effects of hydroxychloroquine.

## Introduction

L´hydroxychloroquine est un antipaludéen de synthèse utilisé dans de nombreuses pathologies comme le lupus, la dermatomyosite, le syndrome de Gougerot-Sjögren, la porphyrie ou encore la sarcoïdose. Les principaux effets secondaires sont oculaires, hématologiques, cardiaques, digestifs et cutanés [1]. Nous rapportons un cas rare d´exanthème maculo-papuleux apparu une semaine après la prise de l´hydroxychloroquine 400mg indiquée pour le traitement d´un atteinte articulaire dans le cadre d´un syndrome de Gougerot-Sjögren primaire.

## Patient et observation

Notre patiente de 41 ans a eu comme antécédent une lésion glomérulaire minime idiopathique déjà traitée efficacement avec la corticothérapie per os. Elle s´est présentée à notre consultation en mars 2019 pour une polyarthralgie inflammatoire des grosses et petites jointures évoluant depuis 3 mois associée à une xérostomie, une xérophtalmie. L´examen ostéoarticulaire a objectivé des arthrites des épaules et mains. La biopsie des glandes salivaires accessoires a mis en évidence une sialadénite lymphocytaire chronique diffuse de grade 4 selon Chisholm et Mason, sans granulome épithélioide et gigantocellulaire ni dépôts amyloïdes ni signe de malignité.

Le test de Schirmer était significatif à 3mm et l´anti-SSA positif à 2,10. Les anticorps antinucléaires (AAN), anti-SSB, anti-DNA, anti-Sm, anti-CCP, facteurs rhumatoïde étaient normaux. Les sérologies hépatites virales B et C, HIV, syphilis étaient toutes négatives. La créatinine à 60 μmol/l, l´urée 0,21g/l, l´hémogramme ainsi que les bilans hépatiques n´ont pas objectivé d´anomalies. L´électrophorèse plasmatique des protéines était normale, la protéinurie de 24 heures négative, l´examen cytobactériologique des urines (ECBU) était stérile sans hématurie ni leucocyturie. Finalement, le diagnostic de syndrome de Gougerot-Sjögren primaire a été retenu. La tomographie en cohérence optique (OCT) maculaire, le champ visuel automatisé et le fond d´œil étaient normaux. La patiente a été mise sous hydroxychloroquine à la dose de 200mg x 2/jr, Salagen 5mg/jr et des larmes artificielles à la demande.

**Figure 1:**
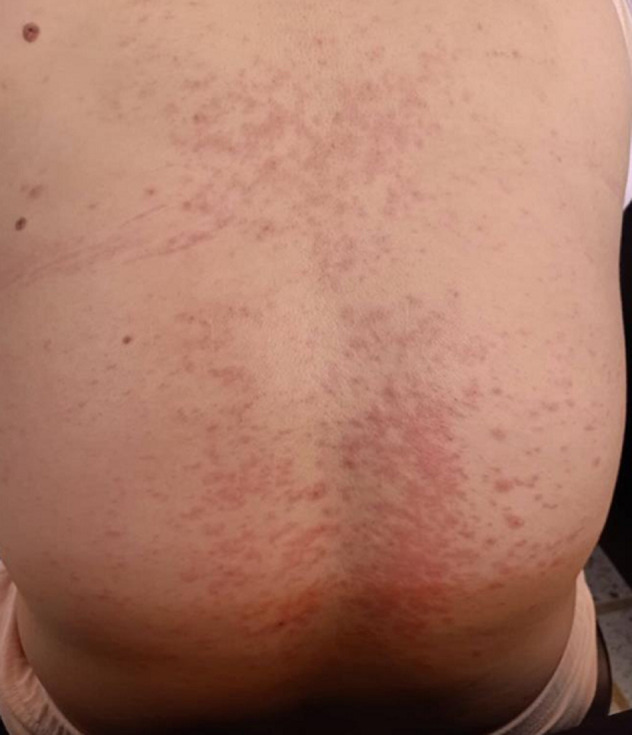
exanthèmes maculo-papuleux au dos

**Figure 2:**
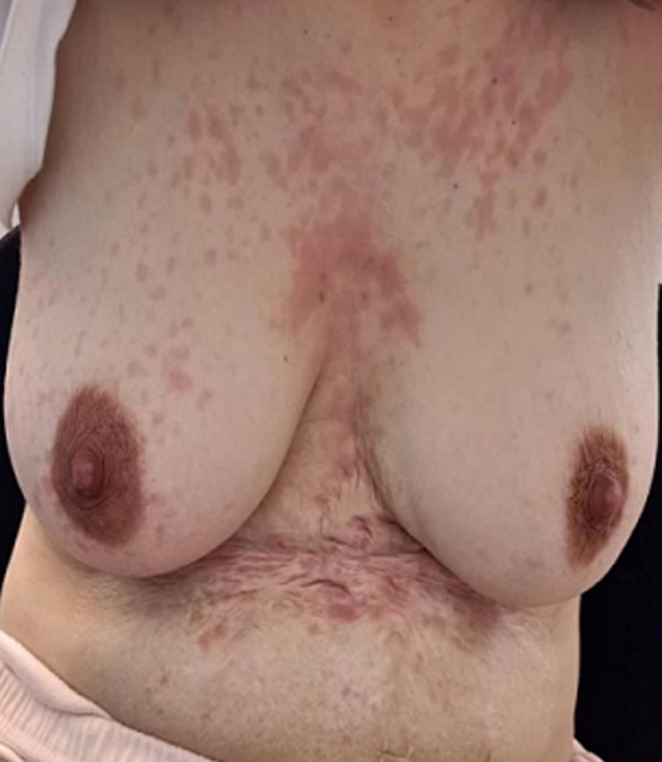
exanthèmes maculo-papuleux au tronc

Après une semaine de prise d´hydroxychloroquine, la patiente a présenté soudainement un prurit diffus avec des macules érythémateuses, papules au tronc et au dos (Figure 1 et Figure 2) sans atteintes des muqueuses ni fièvre ni ictère associés. Il n´y avait pas de notion d´épisode antérieur ni de photo-exposition prolongée ni de prise de nouveaux médicaments ou aliments allergènes. La numération formule sanguine (NFS) était sans particularité en dehors d´un taux de polynucléaires éosinophiles à 550/mm^3^. Les bilans hépatiques, rénaux et thyroïdiens étaient normaux ainsi que les sérologies hépatites virales B et C, parvovirus, cytomégalovirus (CMV) étaient toutes négatives.

Une biopsie cutanée réalisée a objectivé des petits foyers de spongiose avec présence de nombreux lymphocytes, de rares kératinocytes apoptotiques associées à des phénomènes de vacuolisation dans la couche basale. Le derme papillaire était le siège d´un infiltrat plus volontiers périvasculaire constitué de cellules mononuclées associées à des polynucléaires éosinophiles. La patiente a reçu l´hydrocortisone 100mg IV et la desloratadine 5mg per os avec arrêt immédiat de l´hydroxychloroquine. L´évolution a été marquée par la régression du prurit et la disparation progressive des exanthèmes maculo-papuleux.

## Discussion

L'hydroxychloroquine est un médicament immunomodulateur utilisé depuis 60 ans pour traiter le paludisme et les maladies auto-immunes. De nouveaux usages et avantages potentiels continuent à en émerger. Les préoccupations relatives à la toxicité ont été traitées avec des recommandations de prescription mises à jour. Il est recommandé d´utiliser prudemment l´hydroxychloroquine chez les sujets ayant des pathologies préexistantes rénales, maculaires, prise prolongée de l´hydroxychloroquine et les femmes qui prennent concomitamment le tamoxifène [2, 3].

L´hydroxychloroquine est pourvoyeuse de nombreuses réactions d´hypersensibilité immédiates ou retardées. Les hypersensibilités retardées sous hydroxychloroquine sont multiples et de gravité variable. Elles peuvent se manifester par une hyperpigmentation cutanée, des exanthèmes maculo-papuleux, la pustulose exanthématique aigue généralisée, le syndrome de Stevens Johnson, les érythèmes multiformes, la nécrolyse épidermique toxique, le Drug Reaction with Eosinophilia and Systemic Symptoms (DRESS) syndrome. Le délai d´apparition varie entre 1 semaine - 3 mois [1, 4, 5].

Les exanthèmes maculo-papuleux sont apparus chez notre patiente après une semaine de prise de l´hydroxychloroquine pour les atteintes articulaires de syndrome de Gougerot-Sjögren primaire. Cet intervalle d´apparition des exanthèmes maculo-papuleux concorde avec celui rapporté dans la littérature de 4 - 5 jours [6].

Il y a moins d´effets secondaires cutanées sous hydroxychloroquine rapportées dans la littérature au cours de la prise en charge des manifestations systémiques de syndrome de Gougerot-Sjögren. En 2008, Callaly *et al*. [7] a rapporté la première réaction cutanée à type d´une nécrolyse épidermique toxique sous hydroxychloroquine lors du traitement de syndrome de Gougerot-Sjögren. Notre cas se distingue par le type de l´atteinte cutanée (exanthèmes maculo-papuleux) rarement rapportée dans la littérature au cours de traitement de syndrome de Gougerot-Sjögren sous hydroxychloroquine. Nous insistons sur le bénéfice de prescrire à faible dose de l´hydroxychloroquine chez les patients avec des pathologies rénales préexistantes et de faire une recherche régulière des effets secondaires notamment cutanés.

La plupart des réactions cutanées sous hydroxychloroquine rapportées dans la littérature pour prise en charge thérapeutique des maladies systémiques n´étaient pas fatales (Tableau 1) [7-10]. L´évolution était bénigne chez notre patiente avec une bonne réponse au corticoïde, à l´antihistaminique et à l´arrêt immédiat de l´hydroxychloroquine.

**Tableau 1:** cas rapportés dans la littérature des réactions cutanées sous hydroxychloroquine pour traitement des maladies systémiques

Référence	Réactions cutanées sous hydroxychloroquine	Connectivite	Traitement	Evolution
Callay EL [7] 2008	Nécrolyse épidermique toxique	Syndrome de Gougerot-Sjögren	Corticoïde IV	Amélioration
Aisha Lateef [8] 2009	Pusutlose exanthémateuse aigue généralisée évoluant à la Nécrolyse épidermique toxique	Lupus systémique	Corticoïde IV et Immunoglobuline IV	Amélioration
Jae Jeong Park [9] 2010	Pusutlose exanthémateuse aigue généralisée	Dermatomyosite et polyarthralgie	Corticoïde IV et oral	Amélioration
Leckie MJ [10] 2002	Syndrome de Stevens Johnson	Polyarthrite Rhumatoïde	Corticoïde IV et topique	Amélioration
Notre cas	Exanthèmes maculo-papuleux	Syndrome de Gougerot-Sjögren primaire	Corticoïde IV et Anti-histamine	Amélioration

Point de vue de la patiente: la patiente a exprimé sa satisfaction avec la prise en charge après avoir eu une amélioration sous hydrocortisone, desloratadine et l´arrêt immédiat de l´hydroxychloroquine.

## Conclusion

La prescription avec prudence de l´hydroxychloroquine chez les sujets avec des pathologies préexistantes rénales est fortement recommandé afin d´éviter des effets secondaires néfastes notamment cutanés, oculaires, cardiaques et hématologiques. La sensibilisation et l´éducation des patients concernant les effets secondaires de l´hydroxychloroquine aident énormément à améliorer leur qualité de vie.
